# *HOT* mutation screening in human glioblastomas

**DOI:** 10.4155/fso.15.20

**Published:** 2015-11-01

**Authors:** Daniel Krell, Paul Mulholland, Justin Stebbing, Ian Tomlinson, Chiara Bardella

**Affiliations:** 1 Molecular & Population Genetics Laboratory, Wellcome Trust Center for Human Genetics, University of Oxford, Oxford, UK; 2 Department of Medical Oncology, University College London Hospitals, London, UK; 3 Division of Oncology, Department of Surgery & Cancer, Imperial Center for Translational & Experimental Medicine, Imperial College, Hammersmith Hospital Campus, Du Cane Road, London, W12 0NN, UK

**Keywords:** α-ketoglutarate, 2-hydroxyglutarate, brain, glioblastoma, glioma, hydroxyacid-oxoacid transhydrogenase, isocitrate dehydrogenase, metabolism, mutation

## Abstract

**Aims:**

Somatic mutations in *IDH1* and *IDH2* are described in glioblastomas (GBMs). Mutant IDH1 and IDH2 reduce α-KG to D-2HG which accumulates, and is proposed to promote tumorigenesis. HOT catalyzes the conversion of γ-hydroxybutyrate to succinic semialdehyde in a reaction that produces D-2HG. Since increased HOT enzyme activity could lead to an accumulation of D-2HG, coupled with the fact that only a minority of GBMs carry *IDH1/2* mutations and 2HG accumulation has recently been described in *IDH* wild-type tumors, we analyzed a set of GBM samples for mutations in the *HOT* gene.

**Materials & methods:**

We screened 42 human GBM samples for mutations in *HOT*.

**Results:**

No mutations in *HOT* were identified in the 42 GBM samples screened.

**Conclusion:**

Mutations in the coding regions of *HOT* do not occur at an appreciable frequency in GBM.

## Abstract (lay abstract)

Genetic changes in genes called *IDH* have been shown to occur regularly in brain tumors. These changes result in the production of a chemical called D-2HG which accumulates to a high level in cells and is thought to damage normal cells, causing them to become cancer cells. Genetic changes in other genes may also result in the production of D-2HG and cause cancer in the same way as changes in *IDH* do. One such gene is called *HOT*. This study investigated whether genetic changes in *HOT* could be found in brain tumors.

Gliomas are a diverse group of tumors with respect to morphology, genetic status and prognosis and account for 70% of all primary CNS neoplasms. WHO grade II and III gliomas are invasive and typically progress to higher-grade lesions with poor outcome. Glioblastoma (GBM) represents the commonest glioma and may develop in the absence of a less malignant precursor (primary GBM), or through progression from a lower-grade tumor (secondary GBM). GBM carries a very poor prognosis [1,2].

Somatic mutations in genes encoding the enzymes IDH1 and IDH2 have been identified through genome-wide sequencing studies in approximately 70% of grade II and III gliomas, and in approximately 5% of GBMs [3,4], (for a review see [5]). These mutations are early events in gliomagenesis [6], which are associated with a relatively favorable prognosis [7], and may be clinically useful as diagnostic [8,9] and predictive biomarkers [10], as well as potential therapeutic targets in the future [11]. Mutations in *IDH1/2* also occur in patients with acute myeloid leukemia (AML) [12], angioimmunoblastic T-cell lymphoma (AITL) [13] and other solid tumors [14–19].

IDH1 and IDH2 are homodimeric enzymes that catalyze the oxidative decarboxylation of isocitrate to α-KG and reduce NADP to NADPH. IDH1 is localized to the cytoplasm and peroxisome [20], where it serves as a major source of cytosolic NADPH, plays a role in cellular metabolic processes [21,22], and protects against oxidative stresses [23]. IDH2 is localized to the mitochondrion where its main function is to regulate the tricarboxylic acid cycle [20]. A third enzyme, IDH3, is also localized to the mitochondrion and plays a key role in tricarboxylic acid cycle function.

Pathogenic mutations in *IDH1* and *IDH2* are heterozygous somatic missense changes that almost always occur at a single, specific residue at the enzyme active site. Mutations in *IDH1* generally affect arginine 132 (R132) [3], which is commonly substituted for histidine (R132H), but mutations of R132 to serine, cysteine, glycine, leucine and lysine have also been identified [4,24]. Moreover, mutations in *IDH1* occurring at arginine 100 (R100), which may also be pathogenic [19,24–25] have also been identified in gliomas. Mutations in *IDH2* affect arginine 172 (R172) and arginine 140 (R140), the former being analogous to IDH1 R132 and the latter to IDH1 R100 [4,26]. Mutations in the active site arginine residues possess neo-enzymatic activity, which results in the reduction of α-KG to D-2HG [19,26–28], which accumulates at high levels in *IDH1-* and *IDH2*-mutated tumors [26,27]. It has been stated that D-2HG acts as an oncometabolite, with numerous potential protumorigenic effects, most likely as a result of its ability to competitively inhibit α-KG-dependent enzymes [29,30]. Moreover, a direct correlation between the amount of 2HG produced by mutant *IDH1/2* and the characteristics of the resulting tumor seems to exist, since patients with *IDH2* R140-mutant AML produce lower levels of D-2HG and have a better prognosis those with *IDH2* R172 mutations [19].

Out of the 60 or more, α-KG-dependent enzymes in human cells, particular interest has centered on the inhibition by D-2HG of HIF-PHD, the Jumonji-C domain containing histone demethylases, the Ten-Eleven Translocation family of 5-methylcytosine hydroxylases and collagen prolyl 4-hydroxlase. Inhibition of these enzymes could lead to tumor formation by causing HIF1α target gene overexpression [29,31–32], histone modifications [29,33], aberrant DNA methylation [29–30,34–36] and abnormal collagen maturation [29,32], respectively.

D-2HG normally functions as an intermediate in the production of 5-aminolevulinate and porphyrin in heme synthesis [37,38]. Our current understanding of the cellular metabolism of D-2HG is limited, but two enzymes, D-2HGDH and HOT are known to affect its cellular levels [39,40]. In fact, early studies performed on mitochondrial fraction of rat kidney, liver and brain demonstrated that D-2HG is produced from the activity of HOT, which catalyzes the conversion of GHB to SSA, with a stoichiometric production of D-2HG from α-KG [41], (for a review see [42]), and the existence of HOT in humans was then demonstrated in homogenates of human liver, lymphoblast and fibroblasts [43,44]. D-2HG produced by HOT is subsequently interconverted to α-KG via the activity of D-2HGDH, in order to maintain carbon balance.

Homozygous inactivating mutations in *D-2HGDH* lead to D-2HG accumulation in tissues, resulting in the neurological condition hereditary D-2-hydroxyglutaric aciduria (D-2-HGA) [43]. D-2-HGA is associated with developmental delay, hypotonia and seizures, but not an increased risk of brain tumors. Conversely, l-2-hydroxyglutaric aciduria, which occurs due to inactivating mutations in *L-2HGDH*, is weakly associated with the development of brain tumors [45]. Germline *IDH2* mutations affecting arginine 140, have also been reported in patients with D-2-HGA carrying wild-type (WT) *D2HGDH* [46]. However, we and others have not identified *D2HGDH* mutations in gliomas [47,48]. Mass isotopomer studies in patients with the D-2-HGA have demonstrated that accumulated D-2HG derives from mitochondrial α-KG [49], and it has been suggested that alterations in HOT activity may be involved in the excess D-2HG production observed [41,44,50–51]. However enzyme activity assays in fibroblast homogenates from patients with D-2-HGA demonstrated normal HOT activity [44].

Nevertheless, potential evidence for the ability of increased HOT activity to result in excess D-2HG production comes from the observation of patients with the hereditary disorders SSADH deficiency [52], and α-ketoglutarate dehydrogenase deficiency [53]. In addition to GHB, considerably increased levels of D2HG were observed in body fluids of some patients affected by SSADH deficiency, and consequently in this disease elevated intracellular GHB levels are thought to drive HOT enzyme activity to produce secondary increased levels of D2HG [52]. Interestingly, D2HG also accumulates in the brain, liver and kidney of SSADH-deficient mice, showing a consistent link between SSADH deficiency and D2HG accumulation [54]. In patients with α-ketoglutarate dehydrogenase deficiency accumulated α-KG is thought to alter the kinetic equilibrium of HOT [53] and may provide an explanation for the elevated urinary 2HG levels observed in this condition [55,56]. Furthermore, siRNA directed inhibition of HOT function has been shown to reduce cellular 2HG levels *in vitro* [57].

Interestingly, 2HG has recently been shown to accumulate at high levels in a subset of *IDH* WT gliomas, using magnetic resonance spectroscopy [58]. Furthermore, 2HG has been shown to accumulate at millimolar concentrations in *IDH* WT breast cancer tissues, in association with *MYC* gene expression [57]. These findings raise the possibility that mechanisms of 2HG accumulation unrelated to *IDH* mutation may exist in gliomas.

Since an increase in HOT enzyme activity could lead to an accumulation of D-2HG, with tumorigenic effects similar to those observed in tumors with mutant *IDH1/2* and 2HG has recently been shown to accumulate at high concentrations in *IDH* WT tumors, coupled with the fact that only a minority of GBMs carry *IDH1/2* mutations, we analyzed a set of GBM samples for mutations in the *HOT* gene.

## Materials & methods

### Sample collection

All brain tumors were obtained from the neuropathology department at The Royal Free Hospital, Hampstead, London. All 42 samples analyzed were confirmed to be WHO grade IV glioblastoma. We have worked solely on anonymous samples. Study of these has been approved by Oxfordshire REC B 05/Q1605/66.

### DNA extraction

DNA was extracted from paraffin embedded samples using DNeasy Blood and Tissue from QuiagenR (CA, USA), following the manufacturer's instructions.

### Sequencing analysis

Mutation screening of each gene was performed by direct sequencing of genomic DNA in forward and reverse orientations using the Applied Biosystems BigDye terminator reaction kit and the AB 3730xl sequencing machine (Applied Biosystems, CA, USA). Primer sequences were designed to encompass all 14 exons of HOT (RefGene:NM_144650). Primer sequences are illustrated in Table 1. PCR conditions are available on request.

## Results

We screened 42 primary glioblastomas (WHO grade IV) for mutations in the 14 exons of *HOT*. Four of these tumors were known from previous analysis to carry *IDH1* R132H mutations, but none carried mutations in *IDH2, IDH3, L2HGDH* or *D2HGDH* [47]. No somatic mutations were identified in *HOT*. One previously described missense single nucleotide polymorphism (GCC->GTC) was identified in exon 1 of *HOT*.

## Conclusion & future perspective

We conclude that mutations in the coding regions of *HOT* do not occur at an appreciable frequency in IDH WT GBM. The fact that *IDH* mutations occur more frequently in grade II and grade III glioma than in GBM raises the possibility that *HOT* mutations may also be identified in earlier grade tumors than those included in this study.

It is also feasible, however, that although mutations in the coding region of HOT were not identified in the IDH WT GBM patients who were studied, other genetic changes with the potential to increase HOT activity might exist in these tumors. For example, copy number variations encompassing *HOT* could occur, thus increasing gene expression. Indeed interestingly, several large copy number variations have been described encompassing the *HOT* gene, including one linked to AML [59]. Alternatively, mutations in possibly uncharacterized regulatory regions of *HOT*, such as the binding sequence for transcriptional repressors or activators, or epigenetic changes such as alterations of promoter methylation could occur and result in an increase in *HOT* transcriptional activity. Interestingly, promoter hypermethylation affecting *HOT* transcriptional activity has been identified in colorectal cancer, although in this case, this led to a downregulation in *HOT* expression [60].

It remains to be seen if mechanisms of 2HG accumulation unrelated to *IDH* mutation exist in gliomas. However, in view of the fact that 2HG has recently been shown to accumulate in IDH WT tumors, additional studies are required to identify pathogenic mutations in *HOT* and other candidate genes such as *MYC*, which may have the potential to increase D-2HG production and result in cellular effects similar to those seen in *IDH1* and *IDH2* mutant tumors.

Therapies targeting mutant IDH1 and IDH2 have been developed, which have been shown to block D-2HG production and to reverse some of the cellular effects associated with mutant *IDH* expression [61,62]. Furthermore, the targeted inactivation of IDH1 R132H by the inhibitor AGI-5198 has been shown to impair the growth of *IDH1*-mutant glioma cells and mouse xenografts, and promote gliogenic differentiation [63]. Although the potential clinical benefit of such inhibitors is not yet clear, their emergence raises new hope for the treatment of *IDH*-mutated tumors. If mutations in genes other than *IDH1* and *IDH2* are indeed discovered to have the potential to increase cellular D-2HG levels, with protumorigenic effects, then such mutations may also serve as potential therapeutic targets in the future.

**Table 1.** **Primer sequences encompassing the 14 exons of *HOT.***

**Exon**	**Forward primer**	**Reverse primer**
1	TGGCTTGAGGCTTAGACAGG	AAGACTGCGCAGGATCTGA
2	TGCCAAGAACTACCAATTTGA	GCCAAATGCAGAAACCAAAA
3	TGTTCACTGCTGGATTTTACTATCA	TGGTCTGATCAAATAGAGATTATGGA
4	ATGGACTGGCCAACTCTCAA	CTTCTGGCAAACTCACGTTG
5	GCAGTTCTAGTGGAGTTGAACCTA	GCTTCTGCTTAAAGTAAAGAAACAA
6	GGCATGGCAATTTATTTCTGA	CTCATTGCAAAATAAAACAAACA
7	TTTCCTTCTCTAATTTTGTTCCTCA	TGGAGTTGAAAGGTAAGGGAGA
8	AATGATGCCCATGGCTTTAG	TGTGCGTTGAGCAACAAAAT
9	GAGGAAGATGACGCTTTCCA	TGTGGGCCTCCTGTTCTATT
10	AGGAATTGCCAATGTTGATG	AAATTTTACCACCTGTCCTGAA
11	GCATCCCCATAAGAGTATCTTTC	TCTAAGCAGGCGAGAATTGG
12	CAGGGAAGAACAGCTTTGTCA	CTCCCAAAATGCTGGGATTA
13	AGAGCCCGTTTCTTCCTCTC	CTCCTTCGCAGGGAGTGA
14	CTTTTCAAAGCCCTGGGTAA	TCAGCTCTTGCTCTCAGCAC

**Executive summary**

Somatic mutations in *IDH1* and *IDH2* occur in grade II and III gliomas, and primary and secondary glioblastomas (GBMs).
Mutant IDH1 and IDH2 exhibit novel enzymatic activity, reducing α-KG to D-2HG, which accumulates.
D-2HG is thought to act as an oncometabolite through its inhibition of α-KG dependent enzymes.
2HG has recently been shown to accumulate in *IDH* wild-type tumors.
HOT catalyzes the conversion of GHB to SSA in a reaction that converts α-KG to D-2HG.
Mutations in *HOT* could also therefore potentially lead to D-2HG accumulation and thus to tumorigenesis via mechanisms similar to those associated with mutant *IDH1/2*.
We screened 42 human GBM samples for mutations in the coding regions of *HOT*, but none were identified.
Hence, we conclude that mutations in the coding region of *HOT* do not occur at an appreciable frequency in GBM.
However, somatic copy number alterations and epigenetic changes such as altered promoter methylation may also have the potential to alter HOT activity in glioma.
Additional studies are warranted to identify pathogenic mutations in *HOT* and other genes which may have the potential to increase D-2HG production and result in cellular effects similar to those seen in *IDH1* and *IDH2* mutant tumors.
